# Incidental Cyst in an Explanted Liver

**Published:** 2011-08-01

**Authors:** B. Geramizadeh, N. Omidifar

**Affiliations:** *Department of Pathology, Transplant Research Center, Shiraz University of Medical Sciences, Shiraz, Iran*

A 26-year-old young man underwent orthotopic liver transplantation (OLT) for primary sclerosing cholangitis (PSC). He was a known case of PSC since one year before. During this period, he developed jaundice and pruritus and also bloody diarrhea. Colon biopsy was in favor of ulcerative colitis. His laboratory tests showed high serum ALT and AST activities and very high level alkaline phosphatase and GGT. CT of the liver depicted a hypo-attenuated cyst in the right anterosuperior part of the liver, with the possibility of infection or abscess formation.

The explanted liver was large and green. Serial cut sections of the liver showed dilated biliary ducts, some of which contained bile sludge. One of the slices revealed a cystic structure with thin membrane (Figure). 

**Figure:**
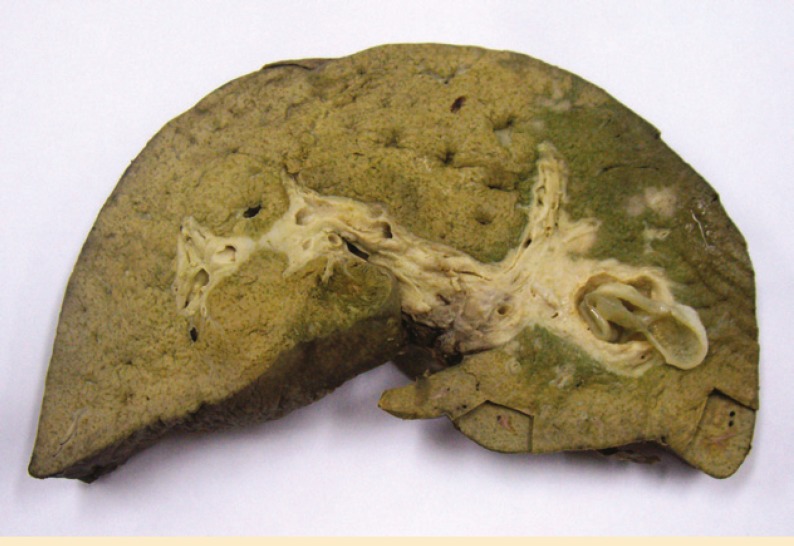
Slice from explanted liver with primary sclerosing cholangitis show an intrabiliary duct cystic structure

**WHAT IS YOUR DIAGNOSIS?**

**DIAGNOSIS: INCIDENTAL LIVER HYDATID CYST IN AN EXPLANTED LIVER WITH PSC**

Hydatid cyst, caused by *Echinococcus granulosus* larva, most commonly can be found in countries of the Middle East, Eastern Europe, Africa, Latin America and China [1].

The most common sites of infection are liver (59%–75%) and lung (27%) [1]. Incidental finding of hydatid cyst has been reported from countries such as Iran, Pakistan, and other endemic countries in unusual locations such as adrenal [2], kidney during nephrolithotomy [3], appendix [4], *etc*.

To the best of our knowledge, no case of incidental hepatic hydatid cyst has been reported after liver transplantation. After this incidental finding thorough investigation of the past history of the patient we found that he grew up in a farm in Ardebil province in close contact with sheep. In the pre-operative CT, the cyst was detected; however, because of its intra-biliary duct location, it was incorrectly diagnosed as a bile duct dilation secondary to PSC. Fortunately, the cyst was intact and the patient did not develop any complications. It is worthy to note that all serologic tests for hydatid cyst performed after OLT were negative.

As a conclusion, in endemic areas of the world hydatid cyst should be considered in the differential diagnosis list of any cyst of the liver.
